# Development of Dual Drug Loaded Nanosized Liposomal Formulation by A Reengineered Ethanolic Injection Method and Its Pre-Clinical Pharmacokinetic Studies

**DOI:** 10.3390/pharmaceutics10030151

**Published:** 2018-09-06

**Authors:** Muhammad Sarfraz, Attia Afzal, Tan Yang, Yongkang Gai, Shahid Masood Raza, Muhammad Waseem Khan, Yao Cheng, Xiang Ma, Guangya Xiang

**Affiliations:** 1 School of Pharmacy, Tongji Medical College, Huazhong University of Science and Technology (HUST), Wuhan 430030, Hubei, China; chiefpharm@gmail.com (M.S.); attiapharm@gmail.com (A.A.); yangtan0120@163.com (T.Y.); gykmail@gmail.com (Y.G.); shahipharmacist@gmail.com (S.M.R.); khanwaseem6065@gmail.com (M.W.K.); cheng_yao@wuxiapptec.com (Y.C.); 2 International Joint Laboratory of Nuclear Protein, Henan University, Kaifeng 475001/475004, Henan, China; 3 Faculty of Pharmacy, The University of Lahore (UOL), Lahore 56400, Punjab, Pakistan; 4 Institute of Pharmacy, Lahore College for Women University (LCWU), Lahore 54610, Punjab, Pakistan

**Keywords:** oleanolic acid, doxorubicin, pegylated liposomes, reengineered ethanolic injection method, cardiotoxicity

## Abstract

Oleanolic acid (OA), which is a natural pentacyclic terpenoid, has been identified for hepato-protective, nephron-protective and cardio-tonic properties. In contrast, doxorubicin (DOX) is a famous anti-cancer drug but its efficacy is a question mark because of its known cardio-toxicity. We developed a combined nanoliposomal formulation of DOX with OA, as adjuvant, to overwhelm toxic effects of DOX without compromising anticancer activity. The entrapment efficiency and the particle size were brought in limit by the reengineered ethanolic injection method (REIM), without further extrusion. The developed formulations were stable over the study period of two months. A modified HPLC method was employed for the analysis of OA (drug retention time, Tr = 12 ± 1 min). The recovery of OA against spiked plasma samples was more than 90%. MTT assay showed anti-apoptotic synergism against HepG2 cells at non-fixed ratio (combination index, CI < 1). A sustained in vivo drug release of experimental drugs was depicted over 24 h. Histopathological examination and laboratory findings indicated no visible sign of toxicity in the treated mice group against combined delivery. Hence, this combined nanoliposomal formulation was tagged as a safer therapy for the DOX based cancer treatments.

## 1. Introduction

Combination anticancer therapy has long been in practice among the clinicians with the objectives to slowdown carcinogenesis and/or to get higher therapeutic efficacy with higher target selectivity via synergism [1]. In the meanwhile, multiple drugs can suppress the notorious phenomenon “cancer chemoresistance”, which is one of the major cause of the failure of single drug therapy [2]. However, as this approach getting attention, it is obvious to consider clinical issues, such as choice of drugs, dose adjustment, and mechanisms of synergy in account to identify the most effective combination treatment regimen [3].

Doxorubicin (DOX), which is an anthracyclineanticancer agent, has been widely used in various chemotherapeutic regimens to treat cancer patients [1,4]. The clinical use of DOX in combination with other cytotoxic drugs recently has increased to get aforesaid advantages, but its associated cardio-toxicity always put a question mark on the pharmacological outcomes. There is increasing interest in strategies to gain higher efficacy with higher safety margin in DOX based chemotherapeutic combinations. One approach with enormous potential is chemoprevention, which is defined as the use of natural, synthetic, or biological agents to reverse, suppress, or prevent carcinogenic progression to invasive cancer [5]. Therefore, a combination therapy of anticancer drug with natural compound is considered to be a promising approach to reduce the chance of reoccurrence of cancer due to chemoresistance developed by the repeated use of anticancer drugs [6,7]. In fact, combination therapy is in agreement that almost all cancer treatments that are in practice utilize multiple drug regimen [8]. Oleanolic Acid (OA, 3β-hydroxyolean-12-en-28-oic acid), a natural pentacyclic terpenoid with known anti-apoptotic activity via interrupting multiple cell signaling pathways, [9] has been reported as hepatoprotective and cardiotonic agent as well [10,11]. Moreover, cellular anticancer activities of oleanolic acid and ursolic acid, a sister compound of oleanolic acid, were reported on various cell lines, such as HepG2, Hep3B, Huh7, and HA22T cell lines [12]. A recent study demonstrated that oleanolic acid β-lactams (OA derivative) showed significant anti-cancer results on HeLa, KB, MCF-7, and Hep-G2 cells as well [13]. Our laboratory reported previously that pegylated liposomal delivery of ursolic acid showed significant anticancer results on KB cell line [14]. Based on above mentioned findings, we were interested to figure out anticancer synergism on selected cell line i.e., HepG2 and KB, while OA and DOX employed in a single carrier system.

Based on the hydrophilic property of DOX and the lipophilic property of OA, the liposomal delivery system was considered a suitable choice for simultaneously delivering both DOX and OA at the target site. It is well understood that the hydrophilic drugs are located in the aqueous core, while the lipophilic drugs are located in the hydrophobic space in the lipid bilayer of the liposomes. On the other hand, liposomes have been using objectively to improve therapeutic index of a range of drugs by ameliorating toxicity and/or synergizing therapeutic effects of the encapsulated agent [15,16].

However, co-delivery of multiple therapeutics is always a challenging task. Alteration of the bilayer barrier properties of the liposomes by the integration of hydrophobic drug may not only affect the penetration of hydrophilic drug in the inner aqueous core but also may interfere its in vivo release at target site. In the meanwhile, inverse correlation between the media ionic strength and the drug encapsulation efficiency was another challenge to control the entrapment efficiency of both drugs at optimum level to get desired therapeutic results [17]. Thin film hydration method followed by extrusion is an efficient technique to develop nano sized liposomes, however, the size homogeneity of the liposomes is negatively impacted after extrusion [18]. Furthermore, In order to minimize the liposome size by extrusion, multiple extrusion cycle with influence of high trans-membrane pressure was required. Ethanolic injection method is a simple, fast, and reproducible technique for the production of a ready-to-use liposome suspension [19]. Here, we employ reengineered ethanolic injection method to incorporate DOX and OA in the liposome without compromising particle size, entrapment efficiencies of both drugs, and drug release, in vitro and in vivo. Various formulation and process variables were also taken in account during the fabrication of both drugs in a single liposomal formulation by reengineered ethanolic injection method (REIM), to see their net impact on colloidal system. The developed formulations were further tested to see in vivo toxicities. Aside by fabrication of dual drug loaded liposomes, a modified validated HPLC method was also developed to quantify the OA in vitro and in vivo samples.

## 2. Materials and Methods

### 2.1. Materials

DOX-HCl (Beijing Hua Feng United Technology Co., Beijing, China), OA (Aladdin Industrial Corporation, Shanghai, China), HSPC (Shanghai Advanced Vehicle Technology Ltd., Co., Shanghai, China), CHOL (Acros Organics, NJ, USA) DSPE.PEG_2000_ (Avanti Polar Lipids, Inc., Alabaster, AL, USA), Sephadex G-25 (GE Healthcare Bio Sciences AB, Uppsala, Sweden) Sepharose CL-2B (Beijing Solarbio Life Sciences, Beijing, China), PBS and HEPES (Biosharp, Anhui, China), CHCl_3_, ethanol, methanol and (NH_4_)_2_SO_4_ (AR grade) and tween 80 (CP grade) (Sinopharm Chemical Reagent Co., Shanghai, China), ACN and MeOH (HPLC grade) (Thermo Fisher Scientific, Geel, Belgium), TFA (AR) (Merck, Darmstadt, Germany) MTT, DMSO and DMEM (Sigma-Aldrich Chemical Co., St Louis, MO, USA), FBS (Zhejiang Tianhang Biological Technology Co., Ltd., Hangzhou, China), DAPI (KeyGen Biotech., Nanjing, China). DMEM (Sigma-Aldrich Chemical Co., St Louis, MO, USA), FBS (Zhejiang Tianhang Biological Technology Co., Ltd., Hangzhou, China), and DAPI (KeyGen Biotech., Nanjing, China).

### 2.2. Cell Culture

HepG2 and KB cell lines were obtained from the China Center for Type Culture Collection at Wuhan University (Wuhan, China) and they were cultured in DMEM medium supplemented with 10% FBS (*v*/*v*), penicillin (100 units/mL), streptomycin (100 mg/mL) under an atmosphere of 5% CO_2_ at 37 °C.

### 2.3. Animals

Female Kunming mice (body weight 18–25 g, 6–8 weeks old) were obtained from Laboratory Animal Center of the Huazhong University of Science and Technology (Wuhan, China). In vivo experimental work was carried out under the outlines of Ethics Committee of Huazhong University of Science and Technology (No. 303). The Kunming mice were kept under ordinary animal care facility of Huazhong University of Science and Technology and were provided with water and food ad libitum till the commencement of bio-study.

### 2.4. Preparation of Liposomes

Briefly, the liposomal formulation, individual and in combination, of model drugs were prepared by TFH method that was further reengineered to ethanolic injection method to maximize drug loading with efficient entrapment of both drugs, single and combined.

#### 2.4.1. Thin Film Hydration Method (TFH)

Briefly, HSPC, CHOL, and DSPE.PEG_2000_ were used in molar ratio of 57:38:5 (~73 g), respectively. All of the lipids were weighed accurately and were dissolved in CHCl_3_ in a 250 mL round bottom flask_._ A dried thin film of the lipids was obtained after evaporation of CHCl_3_ under vacuum at 48 °C by rotary evaporator. This operation was continued for 1 h to remove traces of CHCl_3_. The lipid film was hydrated with 5 mL of aqueous medium (10 mM HEPES/250 mM (NH_4_)_2_SO_4_) and the round bottom flask was continued in rotation for 30 min more at 60 °C without vacuum to get the heterogeneous dispersion of giant and large unilamellar vesicles. This dispersion was subjected to Lipex extruder ((Northern Lipids Inc., Vancouver, BC, Canada)) through 200 nm pore size polycarbonate membrane for five times followed by 100 nm for two times to get final small unilamellar vesicles.

The OA was dissolved in CHCl_3_, along with lipids, while the DOX was loaded by active loading through ammonium gradient to prepare drug loaded liposomes. Briefly, the empty or OA loaded liposomes were prepared first in 250 mM of (NH_4_)_2_SO_4_, as described above_._ The liposomes then subjected to the column filled with Sephadex G 25 to replace the extra vesicular ammonia with dispersion medium (10 mM HEPES, pH 7.2) to establish transmembrane pH gradient for active loading [20]. Then, DOX solution (10 mg/mL in HEPES buffer) was incubated with these liposomes at 60 °C for 90 min. The free DOX was removed by gel filtration (CL-2B gel).

#### 2.4.2. Reengineered Ethanolic Injection Method (REIM)

The employed TFH method was reengineered to ethanolic injection method to develop nanoliposomal formulations of the experimental drugs. Liposomes were prepared with various molar ratio of HSPC and CHOL i.e., 57:38, 64:31, and 38:57, respectively, while the DSPE.PEG_2000_ ratio was kept constant i.e., 5 in all the formulations. The obtained thin film of the lipids was redissolved in 2 mL of preheated absolute ethanol (48 °C). This ethanolic lipid solution was then injected with 1 mL injection syringe to the middle of a 5 mL aqueous phase (10 mM HEPES/250 mM (NH_4_)_2_SO_4_), while the stirring and the temperature (48 °C) were maintained as constant through the experiment for fixed time duration (45 min). The ethanol with some content of water was then removed by rotary evaporation under reduced pressure to get final dispersion, followed by 30 min sonication to get small multi-lamellar vesicles. The OA was dissolved in ethanol to get OA loaded liposomes while the DOX was loaded by active loading through ammonium gradient, as described in “TFH” method above.

### 2.5. Method Optimization

The developed REIM was optimized against various factors to see their impact on physicochemical behavior of liposomes that were discussed below. A combined formulation (ODL), loaded with lipophilic (OA) and hydrophilic (DOX) drugs, was considered as model formulation to address the effects of these factors on PS and EE.

### 2.6. Characterization of Liposomes

The particle size distribution and zeta potential were carried out by a Nano Brook Zeta PALS (Brookhaven Instruments Corporation, Holtsville, NY, USA), based on the dynamic light-scattering principle technique. Each experiment was repeated thrice.

Morphological changes before and after drug loading were observed under transmission electron microscope (TEM, Hitachi, Tokyo, Japan). A drop of the prepared formulation was placed on to a carbon coated copper grid, thus forming a thin liquid film. The excess solution was removed with a filter paper and then the samples were dried in the air before TEM observation.

### 2.7. Determination of Drug Loading (DL) and Entrapment Efficiency (EE)

The entrapment efficiency of the liposome was evaluated after removal of free drug. The following equations were used for calculation:

% Drug loading (% DL) =(amount of encapsulated drug/amount of lipids)×100 % Entrapment Efficiency (%EE) = (amount of encapsulated drug/amount of drug fed) ×100 

DOX concentration in the liposome was measured by UV-VIS spectrophotometer (Spectrum 756PC, Wincom Company Limited, Changsha, China) with λ_max_ = 480 after lysis the liposomes by the appropriate quantity of methanol. Before quantifying the drug in the liposome, the free DOX was removed by passing the liposomes from Sepharose CL-4B/CL-2B column gel package. A standard curve of the DOX in methanol was constructed against concentration range 0.8–25.6 µg/mL.

OA concentration was determined by newly developed HPLC method (Supplementary Data). Validation of the developed method was performed under the International Conference on Harmonization guidelines and Guidance for Industry: Bioanalytical Method Validation [21,22]. The developed method was further validated against spiked plasma samples under strict compliance as we did previously [23,24]. Before lysis the liposome to determine OA concentration, the free OA was separated from the OA encapsulated liposomes. Briefly, the prepared liposomes were subjected to centrifugation (Heraeus Multifuge X1R, Thermo Fisher Scientific, Im Steingrund, Dreieich, Germany) at a low speed of 3000× *g* for 10 min. The supernatant containing OA encapsulated liposomes was separated from free OA pellet in the bottom. A standard curve of OA in methanol was constructed against range 5–160 µg/mL.

### 2.8. Stability Studies of Liposomes

Serum stability was employed by a well-established serum induced leakage assay [25]. Briefly, liposomes in complete DMEM with 10% of fetal bovine serum were incubated by up to 24 h at 37 °C [26]. Time dependent leakage of liposomal DOX was quantified by fluorescence measurements of serum samples by the equation that is given below:

$$\;\%\;DR\;=(\left(F_{t\;}-\;F_{0}\right)/\left(F_{max\;}-F_{0}\right))\times 100\;$$

where, *F*_0_, *F_t_*, and *F_max_* are the fluorescence intensities prior to add liposome (0 time), at specific time interval (*t*) and maximum, while breaking the liposomes by methanol, respectively. Fluorescence intensity measurements were performed using a fluorescence spectrophotometer (F-2700, Hitachi, Tokyo, Japan). The excitation and emission wavelength for DOX were 485 and 585 nm, respectively. Triplicate samples were measured. A calibration curve was constructed against known concentrations (20–500 ng/mL) of DOX in DMEM media [27].

The stability study of OA in serum was employed by the dialysis bag (Mol. Wt. cut off 3500 Da) containing concentrated liposomes (OA conc. 7 mg/mL) (Spectrum™ MicroKros Hollow Fiber Modules, Fisher Scientific, Rancho Dominguez, CA, USA). Briefly, the dialysis bag was immersed in 60 mL of 1% tween 80 in complete DMEM at 37 °C with a rotational speed of 300 rpm. At the different time intervals, the OA content in the dissolution medium was determined by the developed HPLC method. A calibration curve was constructed against known concentrations (5–160 µg/mL) of OA in DMEM media.

### 2.9. Cell Viability Study

Anti-cancer effects of OA and DOX as free solutions and in liposomal formulations were evaluated against HepG2 and KB cells. Briefly, 100 µL of these cells were seeded in 96-well plates at a density of 14 × 10^3^ cells per well and grown for 24 h at 37 °C in a 5% CO_2_ atmosphere. Then, the cells were treated with various concentrations of DOX and OA in 200 µL of sample per well. Culture medium was taken as blank control. After 72 h of incubation, 20 µL of MTT solution (5 mg/mL) was added to each well and cultured for an additional 4 h at 37 °C in a 5% CO_2_ atmosphere. After the media were removed, the blue formazan was dissolved in 200 µL of dimethyl sulfoxide, and the optical density value was determined by the microplate reader (Multiskan MK3; Thermo Fisher Scientific, Atlanta, GA, USA) at 490 nm. Percentage cell growth inhibition (% INH) was calculated by the following formula:

(1) $$\;\%\;\mathrm{INH}=\left[1-\{(A_{T}-A_{B}\right)/(A_{C}-A_{B})\}]\times 100\;$$

where, *A_T_* = absorbance of treated cells (drug); *A_B_* = absorbance of blank (only media); *A_C_* = absorbance of control (untreated cells).

Inhibit 50% of cell growth (IC_50_) and combination index (CI) were calculated by the median effect method of Chou and Talalay [28] using CompuSyn software (version 1.0.1; CompuSyn Inc., Paramus, NJ, USA). The interactions between OA and DOX were evaluated where synergy, additivity, and antagonism are defined as CI < 1, CI = 1 and combination index > 1, respectively.

Off note: The free OA first dissolved in DMSO and then further diluted with DMEM for MTT assay, while the concentration of DMSO was not exceeded more than 0.4% in final dilution as at this concentration no cell growth interruption was observed.

### 2.10. Cell Uptake Study

Binding and internalization efficiency of OA and DOX (solutions/liposomes) in HepG2 cells were examined by fluorescence microscope. Briefly, cells were seeded in a 12 well plates at the density of 7 × 10^3^ cells per well over night. After, the media were aspired off with 1 mL of drug samples (drug conc. = IC_40_, finally prepared in the complete DMEM), and incubated for 24 h at 37 °C in a 5% CO_2_ atmosphere. Then, the cells were fixed for 10 min with 1 mL of 4% paraformaldehyde (pH 7.4 adjusted with diluted HCl) and washed with PBS twice. DAPI (1 mL) was added at a final concentration of 5 µg/mL in saline and incubated for 10–20 min at room temperature. The excess staining (DAPI) was removed by washing with PBS twice again. To examine the degree of apoptosis and internalization of drugs, the stained cells were observed under an Olympus IX71 fluorescence microscope (PerkinElmer Inc., Waltham, MA, USA).

### 2.11. Bio-Distribution Study

In vivo experimental work was carried out under the outlines of Ethics Committee of Huazhong University of Science and Technology (No. 303). Briefly, the drugs (solution/liposome) were injected as a single intravenous bolus via the lateral tail vein of Kunming mice. A 500 μL of blood sample was obtained from eye after removing eye ball and collected in the heparin-treated tubes at 5 min, 15 min, 30 min, 1 h, 2 h, 4 h, 8 h, 12 h, 24 h, and 48 h after the injection, and then centrifuged (5000 rpm, 5 min) to obtain plasma [29]. Later, the mice were sacrificed and the heart, liver, and kidney were also collected only at 1 h, 4 h, 12 h, 24 h, and 48 h for tissue distribution study. All of the plasma and tissue samples were stored at −20 °C until analysis.

Briefly, a 100 µL sample of plasma was taken in 1.5 mL Eppendorf tube and 400 µL of extraction buffer (0.3 M HCl: Ethanol, 3:7, *v*/*v*) was added. The mixture was vortexed for 5 min followed centrifugation at 12,000 rpm for 5 min. The supernatant was collected and stored at 4 °C for future analysis. Similarly, a 100 mg of isolated tissue was accurately weighed, mixed with the same extraction buffer (4 mL) and then centrifuged (12,000 rpm, 5 min) to collect the supernatant. The collected supernatant was stored at 4 °C for future analysis. The concentration of DOX was determined by fluorescence spectrophotometry. The fluorescence of the supernatant was determined at excitation/emission of 485/585 nm to calculate DOX concentration. For OA analysis, the collected samples were pooled and evaporated to dryness under rotary evaporator followed by nitrogen flux at 60 °C and stored at −20 °C. Before the HPLC analysis, the residue was resuspended in methanol (100 μL, HPLC grade) and then centrifuged at 12,000× *g* for 10 min. The supernatent were collected and subjected to HPLC system to quantify the drug. Prior, to quantify the drugs in matrix system, the standard curves for DOX/OA in blood and tissue were generated by the addition of free DOX/OA with different concentrations to blank plasma/tissue, followed by extraction and quantification. The plasma/tissue samples for standard curves were incubated for 24 h at −20 °C after spiking/soaking for proper induction of the drugs in the matrix.

### 2.12. Toxicity Evaluation

Kunming mice (18–25 g, 6–8 weeks of age) were housed well. To check the possible toxic effect of DOX on vital organs i.e., liver, kidney, and heart, the mice were administered with saline (negative control), free DOX (positive control), and DOX loaded liposomes (single and combined with OA). The mice received a cumulative dose of 16 mg/Kg of DOX via i.v. injection, while the dose of not morethan 7 mg/Kg was administered in a single day to avoid possible death of a mouse due to cardiac arrest. The mice were sacrificed after 24 h of the last injection. Blood samples were collected and then centrifuged at 5000 rpm for 5 min to collect plasma. Then, the plasma was used to determine the ALT, AST, BUN and CR levels by automatic biochemical analyzer (Roche ISE900, Basel, Switzerland). The level of glutathione peroxidase (GSH-Px) was also measured in the cardiac tissue. Briefly, the heart was isolated from the mice body and blotted dry on filter paper before weighed. Thereafter, a 10% homogenate of the heart was prepared in a saline to determine GSH concentration. The reagent kit (Nanjing Jiancheng Bioengineering Institute, Nanjing, China) was used to measure the cardiac marker as described by the manufacturer. Body weight of mice was carefully monitored during experiment. Death, if any, was also recorded.

For histophathological evaluation, the liver, kidney and heart specimens were preserved in 4% PFA and they were examined after hematoxylin-eosin staining (H&E) under Inverted Microscope (Olympus IX71, Life Science Solution, Tokyo, Japan) turned on halogen lamp TH4-200, while the condenser torret of the microscope was set at bright field (BF).

### 2.13. Statistics

All data are presented as means with standard deviation (SD). Statistical analyses were performed using Prism 6.0 (GraphPad Software, La Jolla, CA, USA). A one-way ANOVA was used together with a Tukey post-test to compare all of the treatment groups to each other. For the efficacy studies, a two-way ANOVA analysis together with a Tukey post-test was used to determine the effects between different treatment groups in time.

## 3. Results

### 3.1. Preparation of Liposomes

Briefly, the liposomal formulations of OA and DOX were prepared by TFH method. Before extrusion, giant unilamellar vesicles were observed (particle size, PS > 200 nm) with the entrapment efficiencies, EE < 80% for OA in the single (OA loaded liposomes, OAL) and combined (OA and DOX loaded liposomes, ODL) formulations. Although the higher entrapment efficiency of DOX (EE > 80%) was observed in the single drug formulation (DOX loaded liposomes, DXL), but this EE was reduced to 63.21 ± 4.12% in the ODL formulation (Table 1).

After extrusion, the PS was reduced but still it was >200 nm in case of OAL formulation, while the PS was in the limit of 190–200 nm in the case of ODL formulation (Table 1). In case of DXL formulation, the PS was found within the range of 100–200 nm, before and after extrusion. It was also observed that the entrapment efficiencies of the both drugs were markedly decreased after extrusion, especially for OA (Table 1). The polydispersity index (PDI) was below 0.2 and the zeta potential (ZP) was varied between −18 to −28 for all of the formulations, before and after extrusion.

The developed REIM was optimized against various formulation and process variables to see their impact on physicochemical behavior of liposomes, such as lipid concentration and composition, dispersion medium, injection direction, rate of administration, sonication and temperature, % of ethanol in the final formulation, and effect of tween 80 on texture and stability of the vesicular system.

### 3.2. Method Optimization

#### 3.2.1. Lipid Concentration and Composition

**Figure 1:**
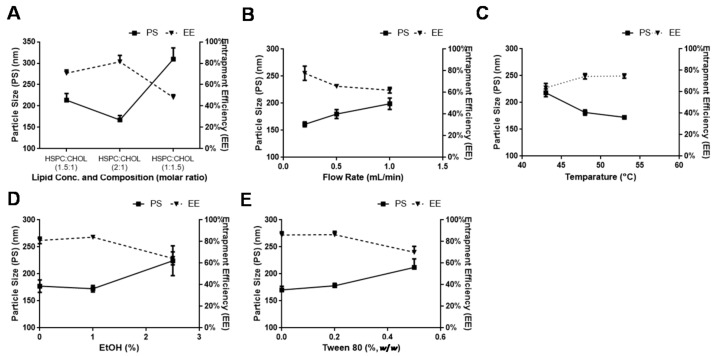
Effect on colloidal system regarding particle size (PS) and entrapment efficiency (EE) of process variables (**A**) Lipid concentration and composition, (**B**) flow rate, (**C**) Temperature, (**D**) Ethanol, (**E**) Tween 80.

Cholesterol (CHOL) is a regulating agent towards liposome membrane fluidity, and it thus improves the drug EE and stability. However, excessive amounts of cholesterol compete for position in the phospholipid bilayer with the lipophilic drug, resulting in decreased EE. Our results were also correlated with this report (Figure 1A).

We also observed a decrease in EE of hydrophilic DOX. In the meanwhile, we revealed that the excessive amount of CHOL, as compared to HSPC, badly affected the particle size as well.

**Figure 2:**
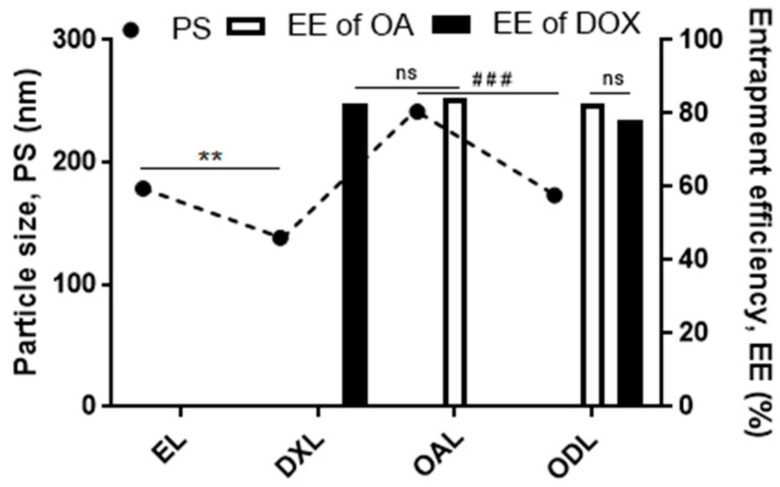
Particle size and EE variation after active loading of DOX at 60 °C. A significant changes were observed in particle sizes of EL and OA loaded liposome (OAL) (*p* < 0.01 (**) and *p* < 0.001 (###), respectively), before and after loading of DOX, while no significant change (ns) was observed in the EE of OA. Similarly, no significant change in EE was observed in the individual loading of both drugs in the liposome.

The loading of both drugs was also accomplished in a single step. Briefly, the OA was dissolved in the organic phase while the DOX was dissolved in the aqueous phase directly. Then, the organic phase was injected to aqueous phase and liposomes were immediately produced, which were manifested by appearance of turbidity in the solution. However, a lower drug entrapment of DOX (EE < 30%) was observed. In order to improve the EE, (NH_4_)_2_SO_4_ medium was used to establish the ammonium gradient to increase the entrapment efficiency of DOX in the liposomes. It was also observed that the active loading of DOX did not affect the %EE of OA (*p* > 0.05), but it caused a significant reduction (*p* < 0.001) in the PS when the DOX solution (10 mM HEPES, pH 7.2) was incubated with OA loaded liposomes for active loading at 60 °C. Similar results were observed when empty liposomes were incubated with the DOX solution for its active loading (Figure 2).

#### 3.2.2. Dispersion Medium

Apparently, solubility and the partitioning property of the drug are the two relevant attributes because of the hydrating/dispersion medium was used for the preparation of liposome [30]. It was observed that the optical density of the OA liposome prepared in HEPES was lesser than the liposome that was prepared in PBS (results not shown).

#### 3.2.3. Injection Direction

Forward injection (organic phase to aqueous phase) and invert injection (aqueous phase to organic phase) were practiced. Forward injection was found practically authentic approach for loading of both drugs because a marked reduction in drug loading of hydrophilic drug (DOX) was observed in the case of invert injection (EE of DOX < 50%).

#### 3.2.4. Rate of Administration

A single fast injection or injection with velocity varied from 0.25 to 1 mL/min was experienced while the lipids composition and the concentration were kept constant. A single fast injection resulted to produce lumps in the formulation that were reduced to a smaller size after applying a powerful vibrating force (Ultrasonic Cleaner, Ningbo Scientz Biotech. Co., Ltd., Ningbo, China). The net particle size after single fast injection was larger than the formulation that was developed after slow injection (result not shown). Optically transparent formulation was observed immediately after slow injection (0.2 mL/min) without any further sonication. As the injection velocity was increased, the formulation was shifted from transparent to opaque apparently. Injection rate inferior to 500 µL/min was resulted in a diluted liposome that might lower entrapment efficiency of water soluble drug (DOX), as reported [31]. In the meanwhile, an increment in particle size was also observed while increasing the velocity of injection up to 1 mL/min (Figure 1B).

#### 3.2.5. Sonication and Temperature Effect

Temperature and sonication effects were compiled against combined liposomal formulation. A high impact on PS and EE was observed after changing temperature and applying sonication. A prominent decrease in PS was observed, along with improved entrapment efficiency, collectively (Figure 1C). In the meanwhile, a gel like consistency was also observed in the primary dispersion when organic phase was injected to aqueous phase, while it was disappeared immediately upon sonication. A sonication was also employed on the retrieved formulation after the removal of ethanol and trace amount of water to get smaller vesicles with uniform dispersion in throughout the system.

During the method development, sonication exactly before and after the ethanol evaporation was the turning point to get an optimum formulation with controlled particle size (PS < 200 nm) and improved entrapment efficiency (EE > 90%) for both drugs.

#### 3.2.6. Effect of Ethanol

For the high solubilizing property of ethanol to dissolve OA, including miscibility property of ethanol with water, the ethanol injection method was the best choice to develop combined formulation of lipophilic (OA) and hydrophilic (DOX) drugs. The ethanolic concentration of 1% in the final liposomal formulation resulted to decrease the particle size and improved the EE but further increase in amount of ethanol hindered the drug loading led to decrease EE, while particle size of the liposome was also increased (Figure 1D). After successful incorporation of the drug, the excessive ethanol was removed by rotary evaporator because of its toxic nature, which may affect the ex-vivo antitumor results.

#### 3.2.7. Effect of Tween 80

Surfactant molecule helps to soften the bilayer membrane to be inserted into liposome phospholipid bilayer membrane. Tween 80 was selected as a surfactant for this study. With and without tween 80, liposomal formulations were prepared. Aqueous phases containing tween 80 at concentrations 0.1%, 0.2%, and 0.5% were prepared and then ethanolic lipid solution was injected into them to prepare liposomal formulations. Other steps were kept constant. At the lower concentration (up to 0.2%), the EE of the drug was improved and somehow particle size was also decreased. But, a further increase in concentration resulted in a decrease in EE, while the particle size was also increased (Figure 1E). In the meanwhile, formulations with tween 80 showed faster increase in particle size than the formulation without tween 80 at longer storage (results not shown).

**Figure 3:**
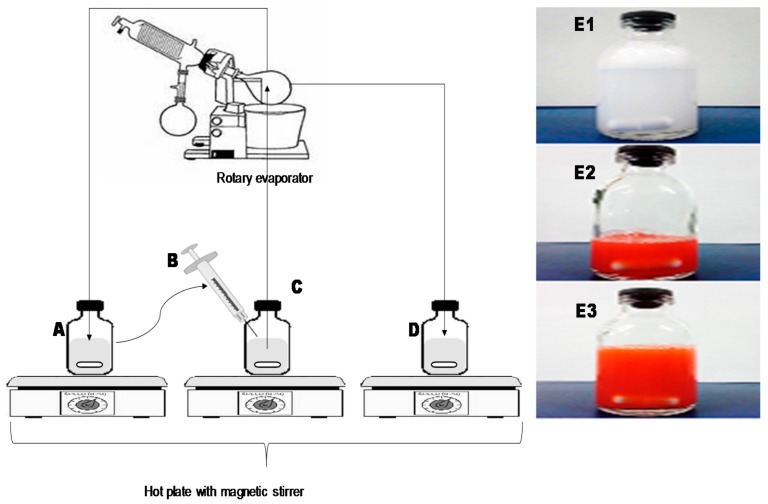
Illustration of reengineered ethanolic injection method (REIM) to produce optimized liposomal formulations, while stirring and temperature (48 °C) were kept constant during various steps (**A**) Dried lipid film was redissolved in ethanol, (**B**) Ethanolic lipid solution was injected to aqueous phase, (**C**) Excess ethanol was evaporated by rotary evaporator, (**D**) Primary liposomal dispersion was collected in a vial and stirred, followed by sonication, (**E1**) Physical appearance of OA loaded liposome (OAL), (**E2**) Physical appearance of DOX loaded liposome (DXL), (**E3**) Physical appearance of combined formulation of OA and DOX (ODL).

Finally, the optimized liposomal formulations produced by the developed REIM were employed for further in vitro in vivo characterization. The results of particle size, surface charges, polydispersity index, and entrapment efficiency are summarized in Table 2. The transparent, elegant, nanoliposomal formulations comprised on unilamellar vesicles were obtained with the particle size range 110–180 nm, while achieving individual drug entrapment efficiency more than 90% in all the formulations (Figure 3).

In contrast to the TFH method, the highest drug was incorporated in the liposome, especially OA i.e., drug to lipid ratio of 1:5 in the OAL formulation, by REIM. Later on, drug to lipid ratio was set to 1:8 to get optimum loading and EE of both drugs in single and combined formulations (Table 2). All of the groups of liposomal formulation were imparted with negative charge. PDI greater than 0.5 was considered a poor formulation with larger particle size.

### 3.3. Characterization of Liposomes

While considering the maximum absorption at 480 nm, UV-Vis spectrophotometry was used to measure the DOX concentration in the quality samples while the drug quantification in matrix system (plasma and tissue) was measured by fluorescence spectrophotometer. DOX concentration in the prepared liposomes was determined by the constructed standard curve (y = 0.0222x − 0.0014, R^2^ > 0.999) against stated range 0.8–25.6 µg/mL (dilutions were prepared in methanol). The OA concentration was determined by the developed validated HPLC method that has already discussed.

**Figure 4:**
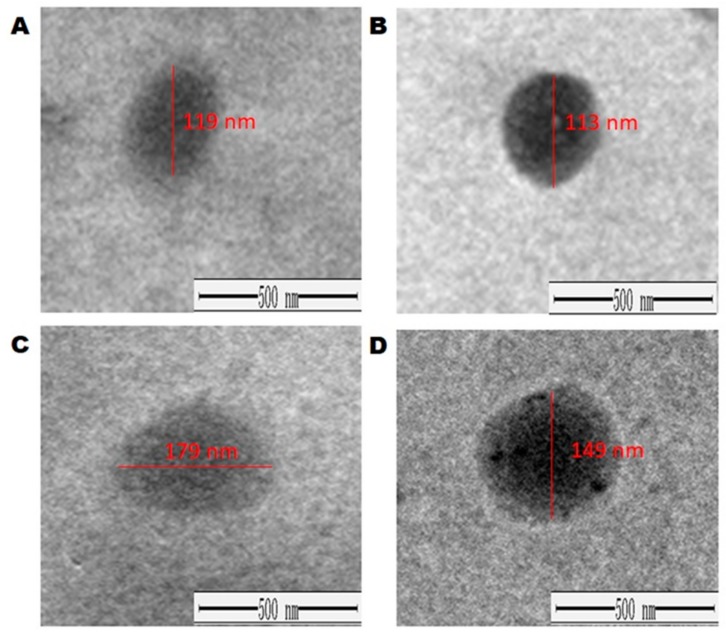
Morphological changes in liposomes before and after loading of drugs under TEM (**A**) Empty liposome (EL), (**B**) DOX loaded liposome (DXL), (**C**) OA loaded liposome (OAL), and (**D**) OA and DOX loaded/combined liposome (ODL).

The mean drug loading efficiencies of DOX were 91.67% and 98%, while the mean EE of OA were 95.67% and 93%, in the single and the combined batches, respectively (Table 2). PDI was satisfactory (PDI < 0.5) and a negative charge was observed on the particles. Morphological changes before and after drug loading were examined under TEM. TEM images confirmed that the vesicles were circular (circularity > 0.9) and the PS was less than 200 nm that is recommended for tumor targeted delivery (Figure 4).

### 3.4. Stability Study of Liposome

**Figure 5:**
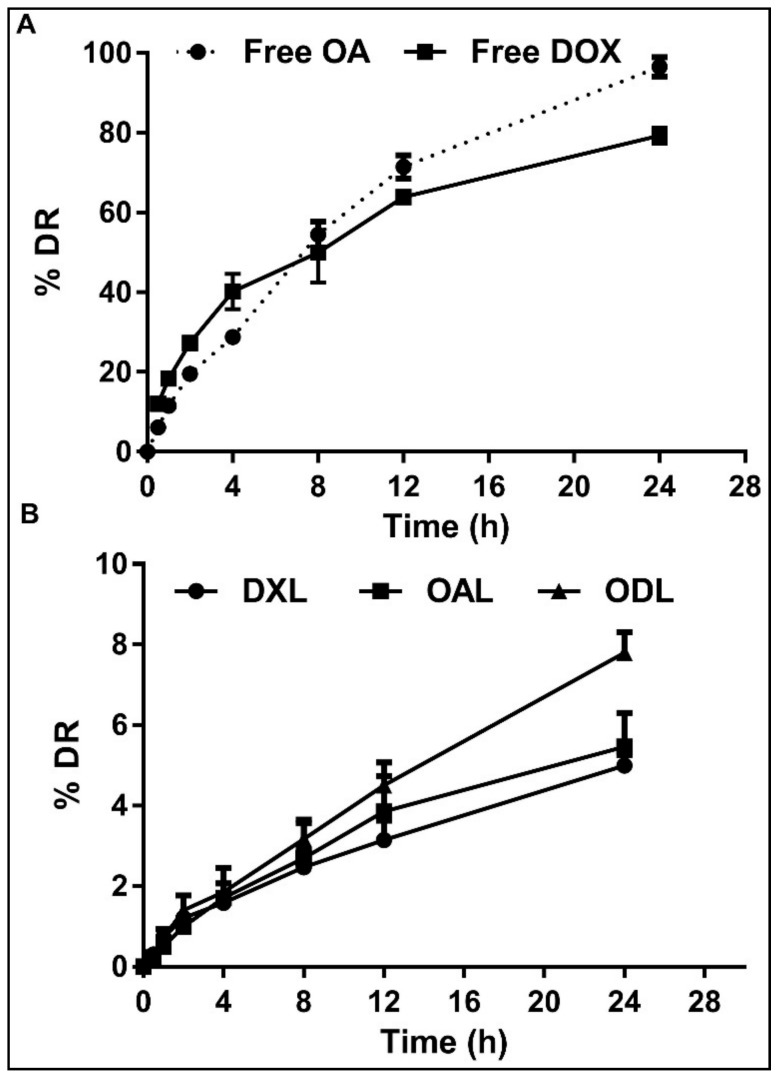
% drug release (%DR) in serum after 24 h (**A**) free drug (**B**) liposome loaded drug.

Our data showed that the developed liposomes were stable in DMEM. The drug release of OA and DOX (single and combined) in serum was in control (~10% only) after 24 h of incubation at 37 °C (Figure 5).

It is reported that cholesterol enhances the affinity of ethanol for the lipid bilayer [32]. So, it might be due to the effect of ethanol integration in phospholipid in the presence of CHOL (as lipids were dissolved in ethanol during preparation) resulting in slow down the release of drugs in serum.

### 3.5. Cell Viability Study

**Figure 6:**
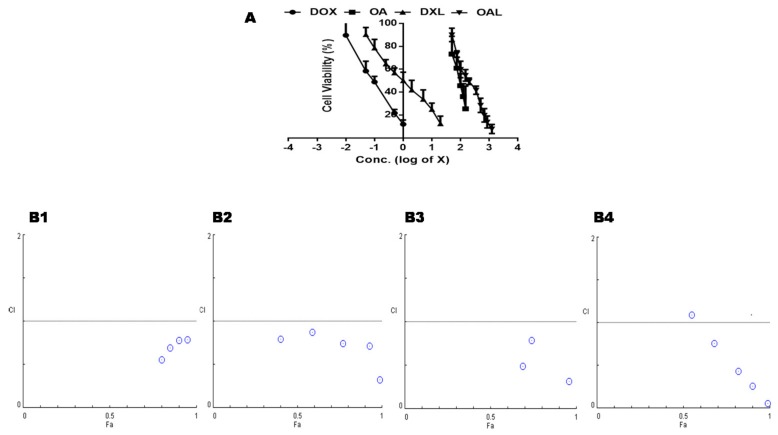
In Vitro antitumor effects on HepG2 cells after various drug treatments; free DOX, free OA, DOX loaded liposomes (DXL) and OA loaded liposome (OAL) (**A**) Concentration dependent gradual decrease in cell viability of HepG2 cells, (**B1**) Combination indices at a fixed concentration of free OA (50 µg/mL)with varying amount of free DOX, (**B2**) Combination indices at a fixed concentration of free DOX (0.05 µg/mL)with varying amount of free OA, (**B3**) Combination indices at a fixed concentration of OA delivered through OA loaded liposome (OAL = 100 µg/mL) against varying amount of DOX loaded liposome, (**B4**) Combination indices at a fixed concentration of DOX delivered through DOX loaded liposome (DXL = 0.5µg/mL) against varying amount of OA loaded liposome.

A conventional approach, MTT Assay, was applied to see the anti-cancer activity of OA, especially in combination with DOX. Cell viability was measured for two cancer cell lines i.e., HepG2 and KB against various drug treatments (single and combined). The concentrations (µg/mL) that were capable to inhibit 50% of cell growth (IC_50_) for DOX was 0.1 ± 0.016 and 0.38 ± 0.05, while IC_50_ was 87.67 ± 15.01 and 189.95 ± 70.94 for OA against HepG2 and KB cells, respectively. Hence, the HepG2 cancer model was considered a better choice for the synergistic anticancer effects of both drugs when employed in the liposomes. The IC_50_ was 1.78 ± 0.27 µg/mL DXL and 181.82 ± 28.22 µg/mL for OAL against HepG2. Based on the concentration dependent cell death of both drugs in a free form/liposome (Figure 6A), the possible synergistic, additive, or agonistic effects were computed on non-fixed ratio of both drugs.

A synergism in anti-apoptotic activity (CI < 1) was observed against HepG2 cells at a fixed concentration of free OA (50 µg/mL) with varying amount of free DOX (0.01, 0.05, 0.1, 0.5, and 1 µg/mL). Similar results were observed for a fixed concentration of free DOX (0.05 µg/mL) with varying amount of free OA (50, 75, 100, 125, and 150 µg/mL) (Figure 6B1,B2).

This synergy was also observed in the liposomal formulation of both drugs when a fixed concentration of liposomal OA (OAL = 100 µg/mL) with varying amount of liposomal DOX (0.05, 0.1, 0.25, 0.5, 1, and 2 µg/mL) was employed on HepG2 cell line. Similar results were also observed when a fixed concentration of liposomal DOX (DXL = 0.5 µg/mL) was used with a varying amount of OAL (100, 150, 200, 350, 500 µg/mL) (Figure 6B3,B4).

### 3.6. Cell Uptake Study

**Figure 7:**
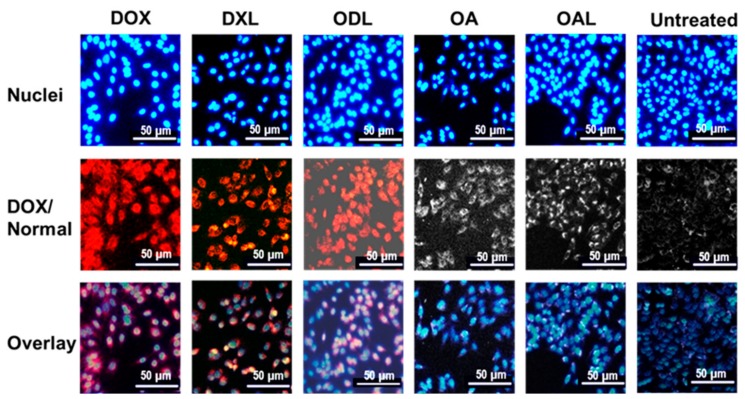
Drug uptake in HepG2 cells after 24 exposure of drug treatments, except DOX (4 h exposure) at drug conc. = IC_40_ under fluorescence microscope (magnification 10×).

Cell uptake study was carried out only on HepG2 cell. The fluorescent property of DOX was used as a measure of its uptake by the cells when it was exposed as free drug or as liposomes (single or combined) under florescence microscope. The induction of apoptosis could be viewed in result of drug binding and internalization that led to enlarged cell nuclei before cell apoptosis (Figure 7). This initial data may lead to the next cellular response of the drug treatments.

### 3.7. Bio-Distribution Study

**Figure 8:**
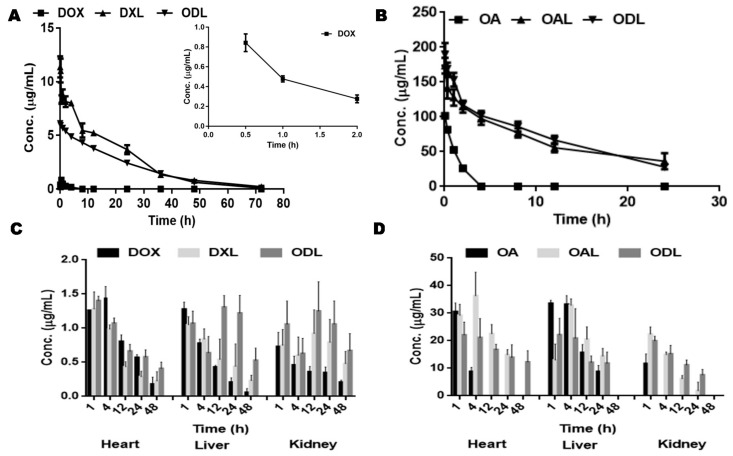
Bio-distribution study of Kunming mice (*n* = 3) after single bolus i.v. injection through tail vein of drug solutions (DOX = 5 mg/Kg and OA = 25 mg/Kg) and liposomes [DOX loaded (DXL = 7 mg/Kg), OA loaded (OAL = 35 mg/Kg), OA and DOX loaded combined, ODL {OA:DOX; 5:1 (OA = 20 mg/Kg, DOX = 4 mg/Kg)}]; (**A**) Plasma vs time profile of DOX delivered through solution or liposomes (**B**) Plasma vs time profile of OA delivered through solution or liposomes (**C**) DOX distribution in vital organs (**D**) OA distribution in vital organs.

Figure 8A, Brepresent the blood clearance profile of DOX and OA after i.v. injection via the tail vein of Kunming mice. The plasma level of free DOX was rapidly decreased at the beginning, as only 1% of the total administered drug was detected after 30 min of drug administration. More than 50% of the DOX was cleared from the body within 30 min after achieving the maximum plasma concentration. This body clearance of free DOX was two-fold faster than free OA. The plasma concentration of both drugs (DOX and OA) delivered through liposomal formulations (single or combined) was significantly higher (*p* < 0.05) than their solutions after i.v. injection, while this higher concentration was persist throughout the study period.

The pharmacokinetics parameters after i.v. injection are listed in Table 3.

In contrast to free drug solution, the half-life (T_1/2_) and mean residence time (MRT) were higher for drug concentrations that were delivered through liposomes, while the clearance (CL) and volume of distribution (V_d_) were significantly lower (*p* < 0.05). The AUC_tot_ were also higher for the drug delivered through liposomes than for drug solutions.

In contrast to the blood compartment, free DOX persisted for a longer period in tissue compartments (4 h for kidney and 12 h for heart and liver) (Figure 8C). It was observed that free DOX deposited more in heart and liver than the kidney compartment. The DOX concentration delivered through liposomes was higher in the heart compartment at initial study period but later decreased gradually. A bell shaped profile (gradually increased and then gradually decreased) of DOX concentrations through liposomes was observed in kidney compartment while a fluctuation in drug concentration was noticed in case of liver compartment, at predefined time points. The observed maximum concentration of DOX (delivered through liposomes) in heart and liver compartments was higher than maximum plasma concentration (C_max_) of that treatment, while the maximum observed drug concentration in kidney compartment was equal to 75% of that plasma C_max_.

A longer residency (24 h) of free OA, after its i.v. administration, was observed in the liver compartment as compared to other tissue compartments. The minimum residing period, only 1 h, of free OA was observed in kidney tissue. The OA concentration that was delivered through liposomes was higher in the initial period and then gradually decreased via tissue compartments throughout the study period (Figure 8D). The maximum concentration of OA delivered through liposomes was observed in the blood compartment, followed by heart and liver compartments.

### 3.8. Toxicity Evaluation

**Figure 9:**
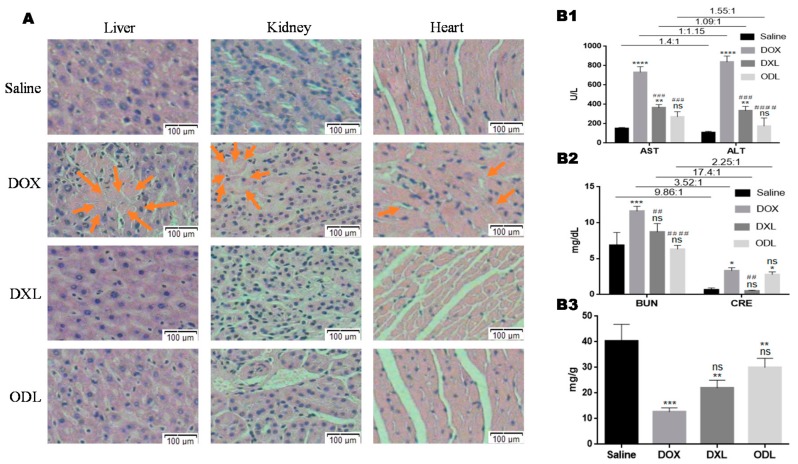
Combined effect of OA and DOX on vital organs i.e., liver, kidney and heart of female Kunming mice (*n* = 3) (**A**) Histopathological examination (H & E staining, magnification: 10×), (**B**) Laboratory findings (biomarkers): (**B1**) Liver function test (AST, ALT), (**B2**), Kidney function test (BUN, CRE), (**B3**) Cardiac function test (GSH-Px). The results compared with control (saline) were represented as: *p* < 0.05 (*), *p* < 0.01 (**), *p* <0.001 (***), *p* <0.0001 (****), and the results compared with DOX were represented as: *p* < 0.05 (#), *p* < 0.01 (##), *p* < 0.001 (###), *p* < 0.0001 (####).

The histopathological results were in agreement of our hypothesis, as we could not find any liver, kidney, and cardiac tissue damage in the ODL treated group (Figure 9A). No unexpected tissue damage sign was also observed in liver, kidney and heart specimens of control group (Figure 9A). However, the heart, the most vulnerable organ against DOX induced toxicity, showed a severe histological damage with a reduction in striated muscle band, including myocytolysis, hemorrhage area, and focal necrosis that were manifested by reducing white spaces between tissues in the DOX treated mice (Figure 9A).

Hepatotoxicity is another side effect of DOX. Severe hepatotoxicity in DOX treated mice was observed, which was evidenced by area of necrosis surrounded by mobilized cells. A marked necrosis in kidney tissue was also observed in the DOX treated group (Figure 9A).

Clinically, the liver and kidney normal function are evaluated based on the biomarker values. The higher ALT value than the AST was a clear indication of liver injury. Hence, the higher AST: ALT ratio (1:1.15) and lower BUN:CRE ratio (3.52:1) for free DOX treated group was the evidence of hepatotoxicity and kidney injury (Figure 9B1,B2). Unlike free DOX, the AST:ALT ratio was observed in limit in the case of DXL treated mice. This was the safety evidence that the liposomal DOX was safer than free DOX for biological system. Similarly, the BUN:CRE ratio for the DXL treated group was within safety window (BUN:CRE between 10–20 is considered an indication of normal function of kidney). Unexpectedly, an alarming indication was received from BUN:CRE ratio for ODL treated group (BUN:CRE < 10 is a laboratory criteria for kidney toxicity) but careful analysis showed that this was basically due to significant difference among CRE value as compared to the control group, otherwise the BUN value was not significantly different than the control group. The other results for the ODL treated group were not significantly different than the control group (*p* > 0.05). Most importantly, the GSH-Px activity (the marker for cardiac function) of the ODL treated mice was close to the normal mice (saline treated mice) (Figure 9B3).

## 4. Discussion

Oleanolic acid (OA) is a pentacyclic triterpenoid compound that exists in the free or bound forms with its sister compound, ursolic acid (UA), in plants, herbs, and other foods. Medicinal use of OA is understood but it rapidly degrades and biologically ineffective in aqueous buffer at physiological pH in the presence of proteins [33]. Moreover, its poor solubility, which leads to decrease bioavailability, reduces its worth to bring in practice [34,35].

Including anticancer, antioxidant, and other pharmacological activities, a novel cardio-tonic potential of OA [10] got attention to use it against DOX induced cardiotoxicity without compromising synergism among anticancer properties of both drugs. The liposome system was selected to fabricate both drugs at fixed ratio that have been shown to be extremely suitable systems to deliver a wide variety of substances to targets of biological, biochemical, pharmacological, and agricultural interest [36,37,38]. Because of a significant role in the thermodynamic stability and concentricity of size distribution of liposomes, pegylated liposomes were preferred to formulate by the developed REIM. On the other hand, it was reported that the ethanol injection method is a prominent technique for scaling-up liposomes production. It offers several advantages, e.g., simplicity, reproducibility, fast implementation, and the fact that it does not cause lipid degradation or oxidative modification and also retained the efficacy of encapsulated drugs as well [39].

The negative charge on the formulations was supposed to be due to the presence of the negatively charged lipid mPEG-DSPE, hence, the high negative potential made the particles repel each other [29].

In the meanwhile, various formulation and process variables were studied to understand their influence on liposome system to design a model formulation containing hydrophilic and hydrophobic drugs, likely OA and DOX, at a fixed ratio with the highest EE and lower PS.

In summary, Cholesterol has recognized effects on liposome: (1) increasing fluidity (2) reducing permeability (3) and stabilizing effect. Moreover, the inclusion of CHOL in liposome also restricted the flexibility of lipid hydrocarbon chains [40], and hence might hinder penetration of hydrophilic DOX, in the liposome during active loading. This tight junction of hydrocarbon chains under the influence of excess CHOL also might be a possible cause of larger particle size. The significant changes in particle size might be due to the effect of temperature (60 °C). With the temperature increased, break of hydrogen bonds and loosening of phospholipids in the bilayer took place and might be the reason of change in the particle size of liposomes. Molarity (ionic strength) and the pH differences among the dispersion media (HEPES and ammonium sulfate) were also considered as other possible reasons of particle size variation. A higher molarity and lower pH (<5.5) of ammonium salt in comparison with HEPES was demonstrated to have an impact on the particle size. The physical appearance in difference in optical density was might be due to differences in the particle size in both the media (PS were 231.67 ± 11.5 and 177 ± 4.58 in PBS and HEPES, respectively). It was also found that DOX was practically insoluble in PBS at pH 7.2, as reported [41]. However, DOX was freely soluble in a 10 mM HEPES at pH 7.2 buffer system. Thus, HEPES buffer system was considered the best choice. In fact, ionic strength of the dispersion medium should be in account when preparing the liposomes because this factor might cause the precipitation of the liposome. However, for the developing self-assembled system, the phenomenon was taken as an advantage to enhance the preparation speed. Furthermore, the net pH of the final formulation depends upon the route of administration, e.g., for i.v. injection, the pH of the dispersion medium containing liposome should be around 7.4 ± 0.2 (as that of blood pH). While injecting aqueous phase to organic phase (invert injection), there was the lesser chance to entrap the aqueous phase during vesicle formation, leading to developing liposomes with smaller inner aqueous compartment. That is the reason that a lesser EE for hydrophilic drug was observed when invert injection was employed (result not shown). It was supposed that fast injection basically reduced the time of interference between organic and aqueous phase and thus resulted to produce loose lumps that were broken on applying high sheer force. Furthermore, a sufficient time of vesicle formation might be not approached because of the fast injection, which might lead to develop liposome with less inner aqueous phase where the key compartment for water soluble drug retained here. Thus, a marked reduction in drug loading/entrapment of hydrophilic drug (DOX) was experienced (EE < 30%). No effect of flow rate was observed on the EE of lipophilic drug (OA) because the lipophilic drug was already entrapped in lipid compartment. Sonication produced many cavitation events in the system, which enhance the dispersion of lipid molecules and therefore reduce the liposome size [42]. Temperature was a universal factor that always showed its influence on the colloidal system whenever it applied. The lipid bilayer membrane fluidity has a profound effect on the particle size of the liposome that is basically targeted by the temperature. Based on this phenomenon, we can expect that, the higher the temperature, the higher the entrapment of the drug, but lower the particle size (Figure 1C and Figure 2). In addition, ethanol, when coming in contact with phospholipids, results in an increase in the dielectric constant, dehydration of the phospholipid head groups, and an increase in ion permeability [43,44,45]. These prominent features of the ethanol made it not only a good vehicle for loading of lipophilic drug, but also for the hydrophilic drug, particularly if the drug is pH dependent based on its ionic strength, namely DOX. Although a promising effect of tween 80 was observed for PS and EE, EE was improved at a lower concentration, but it was decreased at a higher concentration of Tween 80 (0.5%, *w*/*w*) (Figure 1E). Flexible liposomes and surfactant incorporated liposomes sometimes showed less stability than conventional liposomes due to the much increased membrane fluidity. Furthermore, tween 80 was an amphiphilic agent that could form other dispersions, such as micelles, after being released from phospholipid bilayer of the liposome during storage [46,47]. A faster increase in particle size might be the result of this interaction of tween 80 with the dispersion medium at longer storage.

These results helped out to choose the best ratio of lipids to develop optimum formulation, and HSPC, CHOL and DSPE.PEG_2000_ were finally used in molar ratio of 64:31:5.

In parallel of formulation development and characterization, a validated HPLC method for quantification of OA was also developed (Supplementary Data). Moreover, in the best of our knowledge based on literature survey, limited studies reported the quantification of OA in the in vivo samples. We successfully employed our validated HPLC method to quantify OA, not only in the developed liposomal formulation samples, but also quantify the OA in plasma and tissue samples. Inter and intra-day stability results indicated that the developed HPLC method has the potential to use as routine method to quantify the OA in in vitro and in vivo samples.

An effective anticancer synergism was observed at the stated drug combination. In comparison with single treatment, around a 50% reduction in dose of one drug was found to be effective enough to kill 50% of the cancer cells when used in combination with other drug. Hence, the fabricated liposomal combined delivery was suggested as a suitable delivery against HCC. An initial decrease in the first quarter hour and then sudden increase in free DOX plasma concentration after that it decreased fast initially and then gradual elimination from the body was supposed due to enterohepatic circulation, particle size, and the capillary permeability. Furthermore, the fast elimination of free DOX even higher than elimination of free OA might be due to its high hydrophilicity. The higher plasma concentration of investigated drugs (OA and DOX) delivered from liposomes and their lower distribution in tissues as compared to their solutions was already expected because of the longer circulation of pegylated liposomes. Furthermore, the presence of both drugs concentration, delivered through liposomes, in heart tissue till the time of last sampling time point was also in agreement with longer circulation of the pegylated liposomes. The lesser uptake of both drugs, in solutions, by the kidney tissue might be due to continuous blood filtration activity of kidney. The residing of both liposomal drugs in the plasma and tissue compartments throughout the study period, especially at fixed ratio in a combined therapy, might fulfill the objective to deliver the drugs at target site (tumor) in future if loaded the both drugs by developed REIM. A significant difference in concentrations of free and encapsulated drugs was observed (*p* < 0.05) during bio-distribution study.

In contrast of DOX, OA is an effective antioxidant that plays a role in the prevention of multiple diseases that are related to cell oxidative damage. A protective effect of OA against oxidative stress-induced inflammation and cardio-protection activity was also suggested [48]. Hence, based on the above reported evidences and as well as our histopathological results along with laboratory findings, we can express confidently that the OA built a defense line against possible toxic effects of DOX on vital organs (liver, kidney, heart) while administered a combined nanoliposomal formulation of OA and DOX (ODL) to the Kunming mice.

The mechanism, how the OA overcome oxidative stress in the myocardium generated by DOX, is illustrated in graphical abstract.

## 5. Conclusions

A well designed, reengineered, hybrid ethanolic injection method was employed to control entrapment efficiency and particle size. The in vitro anti-tumor study of the developed liposomes substantiated that only toxic effects were detoxified by OA without compromising anticancer activity of DOX, in this combination. Hence, the OA may use in-combination with the other chemotherapeutics to synergize killing effects without compromising the risk of associated cardiotoxicity, if applicable. Moreover, the developed validated HPLC method was employed successfully to quantify OA in the quality and matrix samples. Furthermore, the developed REIM may scale up to develop sustained release stable formulations of two or more drug combination if all of the discussed process and formulation variables are taken in account, properly. The developed HPLC method for OA also may be used as routine analytical method.

In brief, the toxicity evaluation revealed that this combined nanoliposomal delivery of DOX with OA was a safer therapy and may bring in practice after clinical trials.

## Figures and Tables

**Table 1:** Physicochemical attributes of liposomal formulations developed by TFH method (*n* = 3); HSPC:CHOL:DSPE.PEG_2000_ (57:38:5, molar ratio), Drugs:Lipids (1:8, molar ratio), Temp. (48 °C), (OA:DOX, 5:1, *w*/*w*)(mean ± SD).

Batch ID	Before Extrusion					After Extrusion				
	PS	PDI	ZP	%EE_OA_	%EE_DOX_	PS	PDI	ZP	%EE_OA_	%EE_DOX_
OAL	314.33 ± 30.92	0.18 ± 3.06	−18.33 ± 3.06	77.11 ± 3.72		241.33 ± 20.13	0.19 ± 0.02	−18.33 ± 4.16	66.11 ± 3.22	
DXL	155 ± 11.53	0.17 ± 0.06	−27.66 ± 8.96		84.82 ± 2.86	135.33 ± 8.5	0.15 ± 0.03	−19.33 ± 1.53		78.93 ± 1.4
ODL	225.33 ± 28.02	0.19 ± 0.03	−24.33 ± 2.52	70.97 ± 3.80	63.21 ± 4.12	199 ± 10.54	0.15 ± 0.03	−18.33 ± 7.57	63.81 ± 2.25	61.03 ± 1.76

PS: particle size; PDI: polydispersity index; ZP: zeta potential; EE: entrapment efficiency.

**Table 2:** Physicochemical attributes of liposomes prepared by REIM under preset conditions (*n* = 3); HSPC:CHOL:DSPE.PEG_2000_ (64:31:5, molar ratio), Drugs:Lipids (1:8, molar ratio), %Ethanol (0), Temp. (48 °C), OA:DOX (5:1, *w*/*w*) (mean ± SD).

Batch ID	PS	ZP	PDI	%EE_OA_	%EE_DOX_	%EE_Comb._
OAL	127 ± 11.14	−19.33 ± 3.51	0.14 ± 0.07	95.67 ± 3.06		
DXL	136.33 ± 7.02	−11.33 ± 3.05	0.13 ± 0.04		91.67 ± 5.7	
ODL	169.67 ± 16.01	−13 ± 5.3	0.18 ± 0.03	93 ± 2	98 ± 1	95.1 ± 1.5

**Table 3:** Pharmacokinetics parameters after bolus i.v. injection via tail vein of Kunming mice (*n* = 3) against various drug treatments e.g., free drug solutions (DOX and OA) and liposomes [DOX loaded (DXL), OA loaded (OAL), and (combined (ODL)].

PK Parameters	DOX	DXL	OA	OAL	ODL	
					DOX	OA
C_max_ (µg/mL)	0.91 ± 0.12	10.78 ± 1.5	90.83 ± 17.11	172.81 ± 14.72	5.17 ± 0.5	130.74 ± 10.07
AUC_tot_ (µg/mL/h)	1.18 ± 0.15	176.54 ± 9.93	98.83 ± 24.12	2135.24 ± 107.24	124.05 ± 2.68	1846.38 ± 79.35
T_1/2_ (h)	0.6 ± 0.33	12 ± 2.25	0.6 ± 0.14	10.84 ± 1.86	8.49 ± 0.65	8.86 ± 0.55
Vd (mL)	84.69 ± 44.08	16.68 ± 3.53	7.68 ± 0.59	6.93 ± 0.82	16.87 ± 1.12	6.14 ± 0.16
CL (mL/h)	99.02 ± 15.92	0.97 ± 0.17	9.34 ± 2.22	0.45 ± 0.03	1.38 ± 0.03	0.48 ± 0.03
MRT (h)	1.74 ± 0.16	19.11 ± 2.61	0.88 ± 0.16	16.32 ± 2.04	18.2 ± 0.92	13.04 ± 0.82

C_max_: peak plasma concentration: AUC_tot_: Total area under the curve; T_1/2_: half-life; Vd: volume of distribution: CL: clearance: MRT: mean residence time.

## Supplementary Materials

The following are available online at http://www.mdpi.com/1999-4923/10/3/151/s1, Aside by formulation development, the validated HPLC method was also developed for the quantification of OA in the matrix system.

## References

[1] Lee J.H., Nan A. (2012). Combination drug delivery approaches in metastatic breast cancer. J. Drug Deliv..

[2] Hu C.M., Aryal S., Zhang L. (2010). Nanoparticle-assisted combination therapies for effective cancer treatment. Ther. Deliv..

[3] Tallarida R.J. (2001). Drug synergism: Its detection and applications. J. Pharmacol. Exp. Ther..

[4] Minotti G., Menna P., Salvatorelli E., Cairo G., Gianni L. (2004). Anthracyclines: Molecular advances and pharmacologic developments in antitumor activity and cardiotoxicity. Pharmacol. Rev..

[5] Okada S. (2001). Cancer chemoprevention as adjuvant therapy for hepatocellular carcinoma. Jpn. J. Clin. Oncol..

[6] Kojima-Yuasa A., Huang X., Matsui-Yuasa I. (2015). Synergistic anticancer activities of natural substances in human hepatocellular carcinoma. Diseases.

[7] Wang Z.J., Xie C., Huang Y., Lam C.W.K., Chow M.S.S. (2014). Overcoming chemotherapy resistance with herbal medicines: Past, present and future perspectives. Phytochem. Rev..

[8] Tardi P.G., Gallagher R.C., Johnstone S., Harasym N., Webb M., Bally M.B., Mayer L.D. (2007). Coencapsulation of irinotecan and floxuridine into low cholesterol-containing liposomes that coordinate drug release in vivo. Biochim. Biophys. Acta (BBA) Biomembr..

[9] Ziberna L., Samec D., Mocan A., Nabavi S.F., Bishayee A., Farooqi A.A., Sureda A., Nabavi S.M. (2017). Oleanolic acid alters multiple cell signaling pathways: Implication in cancer prevention and therapy. Int. J. Mol. Sci..

[10] Mapanga R.F., Rajamani U., Dlamini N., Zungu-Edmondson M., Kelly-Laubscher R., Shafiullah M., Wahab A., Hasan M.Y., Fahim M.A., Rondeau P. (2012). Oleanolic acid: A novel cardioprotective agent that blunts hyperglycemia-induced contractile dysfunction. PLoS ONE.

[11] Shanmugam M.K., Dai X., Kumar A.P., Tan B.K., Sethi G., Bishayee A. (2014). Oleanolic acid and its synthetic derivatives for the prevention and therapy of cancer: Preclinical and clinical evidence. Cancer Lett..

[12] Yan S.L., Huang C.Y., Wu S.T., Yin M.C. (2010). Oleanolic acid and ursolic acid induce apoptosis in four human liver cancer cell lines. Toxicol. In Vitro.

[13] Zhu Y.Y., Huang H.Y., Wu Y.L. (2015). Anticancer and apoptotic activities of oleanolic acid are mediated through cell cycle arrest and disruption of mitochondrial membrane potential in HepG2 human hepatocellular carcinoma cells. Mol. Med. Rep..

[14] Yang G., Yang T., Zhang W., Lu M., Ma X., Xiang G. (2014). In vitro and in vivo antitumor effects of folate-targeted ursolic acid stealth liposome. J. Agric. Food Chem..

[15] Gregoriadis G. (2016). Liposomes in drug delivery: How it all happened. Pharmaceutics.

[16] Bulbake U., Doppalapudi S., Kommineni N., Khan W. (2017). Liposomal formulations in clinical use: An updated review. Pharmaceutics.

[17] Eloy J.O., Claro de Souza M., Petrilli R., Barcellos J.P., Lee R.J., Marchetti J.M. (2014). Liposomes as carriers of hydrophilic small molecule drugs: Strategies to enhance encapsulation and delivery. Colloids Surf. B Biointerfaces.

[18] Ong G.S., Chitneni M., Lee S.K., Ming C.L., Yuen H.K. (2016). Evaluation of extrusion technique for nanosizing liposomes. Pharmaceutics.

[19] Maherani B., Arab-Tehrany E., Mozafari M.R., Gaiani C., Linder M. (2011). Liposomes: A review of manufacturing techniques and targeting strategies. Curr. Nanosci..

[20] Ye P., Zhang W.D., Yang T., Lu Y., Lu M., Gai Y.K., Ma X., Xiang G.Y. (2014). Folate receptor-targeted liposomes enhanced the antitumor potency of imatinib through the combination of active targeting and molecular targeting. Int. J. Nanomed..

[21] European Medicines Agency Committee for Medicinal Products for Human Use Guideline on Bioanalytical Method Validation.

[22] U.S. Department of Health and Human Services Food and Drug Administration Center for Drug Evaluation and Research (CDER). www.Bioagilytix.Com/wp-content/uploads/2016/02/fda-bioanalytical-method-validation-draft-guidance-2013.Pdf.

[23] Ashraf M., Abid F., Riffat S., Bashir S., Iqbal J., Sarfraz M., Afzal A., Zaheer M. (2015). Rationalized and complementary findings of silymarin (milk thistle) in Pakistani healthy volunteers. Acta Pol. Pharm..

[24] Sarfraz A., Sarfraz M., Ahmad M. (2011). Development and Validation of a Bioanalytical Method for Direct Extraction of Diclofenac Potassium from Spiked Plasma. Trop. J. Pharm. Res..

[25] Evjen T.J., Nilssen E.A., Rognvaldsson S., Brandl M., Fossheim S.L. (2010). Distearoylphosphatidylethanolamine-based liposomes for ultrasound-mediated drug delivery. Eur. J. Pharm. Biopharm..

[26] Alyane M., Barratt G., Lahouel M. (2016). Remote loading of doxorubicin into liposomes by transmembrane pH gradient to reduce toxicity toward H9c2 cells. Saudi Pharm. J..

[27] Evjen T.J., Hagtvet E., Nilssen E.A., Brandl M., Fossheim S.L. (2011). Sonosensitive dioleoylphosphatidylethanolamine-containing liposomes with prolonged blood circulation time of doxorubicin. Eur. J. Pharm. Sci..

[28] Chou T.C., Talalay P. (1984). Quantitative analysis of dose-effect relationships: The combined effects of multiple drugs or enzyme inhibitors. Adv. Enzym. Regul..

[29] Huang Y., Yang T., Zhang W., Lu Y., Ye P., Yang G., Li B., Qi S., Liu Y., He X. (2014). A novel hydrolysis-resistant lipophilic folate derivative enables stable delivery of targeted liposomes in vivo. Int. J. Nanomed..

[30] Nounou M.M., El-Khordagui L.K., Khalafallah N. (2005). Effect of various formulation variables on the encapsulation and stability of dibucaine base in multilamellar vesicles. Acta Pol. Pharm..

[31] Pons M., Foradada M., Estelrich J. (1993). Liposomes obtained by the ethanol injection method. Int. J. Pharm..

[32] Trandum C., Westh P., Jørgensen K., Mouritsen O.G. (2000). A thermodynamic study of the effects of cholesterol on the interaction between liposomes andd ethanol. Biophys. J..

[33] Peng W., Ding F., Jiang Y.T., Peng Y.K. (2014). Bioavailability and activity of natural food additive triterpenoids as influenced by protein. J. Agric. Food Chem..

[34] Fernandez-Hernandez A., Martinez A., Rivas F., Garcia-Mesa J.A., Parra A. (2015). Effect of the solvent and the sample preparation on the determination of triterpene compounds in two-phase olive-mill-waste samples. J. Agric. Food Chem..

[35] Fan J.-P., Kong T., Zhang L., Tong S., Tian Z.-Y., Duan Y.-H., Zhang X.-H. (2011). Solubilities of ursolic acid and oleanolic acid in four solvents from (283.2 to 329.7) k. J. Chem. Eng. Data.

[36] Tan C., Xue J., Abbas S., Feng B., Zhang X.M., Xia S.Q. (2014). Liposome as a delivery system for carotenoids: Comparative antioxidant activity of carotenoids as measured by ferric reducing antioxidant power, dpph assay and lipid peroxidation. J. Agric. Food Chem..

[37] Anderson J.A., Gipmans M., Hurst S., Layton R., Nehra N., Pickett J., Shah D.M., Souza T.L., Tripathi L. (2016). Emerging agricultural biotechnologies for sustainable agriculture and food security. J. Agric. Food Chem..

[38] Nuruzzaman M., Rahman M.M., Liu Y., Naidu R. (2016). Nanoencapsulation, nano-guard for pesticides: A new window for safe application. J. Agric. Food Chem..

[39] Zou L.Q., Liu W., Liu W.L., Liang R.H., Li T., Liu C.M., Cao Y.L., Niu J., Liu Z. (2014). Characterization and bioavailability of tea polyphenol nanoliposome prepared by combining an ethanol injection method with dynamic high-pressure microfluidization. J. Agric. Food Chem..

[40] Jaafar-Maalej C., Diab R., Andrieu V., Elaissari A., Fessi H. (2010). Ethanol injection method for hydrophilic and lipophilic drug-loaded liposome preparation. J. Liposome Res..

[41] Gao X., Wang B., Wei X., Rao W., Ai F., Zhao F., Men K., Yang B., Liu X., Huang M. (2013). Preparation, characterization and application of star-shaped PCL/PEG micelles for the delivery of doxorubicin in the treatment of colon cancer. Int. J. Nanomed..

[42] Huang X., Caddell R., Yu B., Xu S., Theobald B., Lee L.J., Lee R.J. (2010). Ultrasound-enhanced microfluidic synthesis of liposomes. Anticancer Res..

[43] Zeng J., Smith K.E., Chong P.L. (1993). Effects of alcohol-induced lipid interdigitation on proton permeability in l-alpha-dipalmitoylphosphatidylcholine vesicles. Biophys. J..

[44] Rottenberg H. (1992). Probing the interactions of alcohols with biological membranes with the fluorescent probe prodan. Biochemistry.

[45] Chiou J.S., Kuo C.C., Lin S.H., Kamaya H., Ueda I. (1991). Interfacial dehydration by alcohols: Hydrogen bonding of alcohols to phospholipids. Alcohol.

[46] Li C., Zhang Y., Su T.T., Feng L.L., Long Y.Y., Chen Z.B. (2012). Silica-coated flexible liposomes as a nanohybrid delivery system for enhanced oral bioavailability of curcumin. Int. J. Nanomed..

[47] Song J., Shi F., Zhang Z., Zhu F., Xue J., Tan X., Zhang L., Jia X. (2011). Formulation and evaluation of celastrol-loaded liposomes. Molecules.

[48] Sanchez-Quesada C., Lopez-Biedma A., Warleta F., Campos M., Beltran G., Gaforio J.J. (2013). Bioactive properties of the main triterpenes found in olives, virgin olive oil, and leaves of *Olea europaea*. J. Agric. Food Chem..

