# Supplementary material for: Development of Dual Drug Loaded Nanosized Liposomal Formulation by A Reengineered Ethanolic Injection Method and Its Pre-Clinical Pharmacokinetic Studies
Source: Pharmaceutics. 2018 Sep 6;10(3):151. doi: 10.3390/pharmaceutics10030151 (PMC6161256; doi:10.3390/pharmaceutics10030151)
Supplement: Supplementary file 1 [file pharmaceutics-10-00151-s001.pdf]

Supplementary Materials: Development of Dual Drug Loaded Nanosized Liposomal Formulation by A Reengineered Ethanolic Injection Method and Pre-Clinical Studies

Muhammad Sarfraz, Attia Afzal, Tan Yang, Yongkang Gai, Shahid Masood Raza, Muhammad Waseem Khan, Yao Cheng, Xiang Ma and Guangya Xiang

S1. Development of validated HPLC method for quantification of OA

To date, few literatures were found to describe the quantification of OA in the matrix system. Hence, a validated HPLC method for quantification of OA in the quality and the matrix samples was developed.

Briefly, the HPLC system used for quantification of OA consisted of Agilent 1220 Infinity LC modules (Germany), a quaternary pump, mobile phase degasser, auto-sampler with thermostat and a column heater compartment. Agilent software, ChemStation, was used for data acquisition and analysis. The chromatographic conditions were as follows: Agilent Zorbax SB-C18 guard column (2.1 mm × 50 mm); analytical column (4.6 mm × 250 mm, Saphire C18, 5 µm, from Sepax Technologies Inc). OA was detected at a wavelength of 210 nm with a flow rate of 1.3 mL/min. In order to select the best mobile phase, organic solvents such as methanol, acetonitrile in-combination with water (with or without buffer) were used. Various regents such as THF, TFA, TEA were also used in different concentration for the sharp elution of OA. Finally, the mobile phase comprised of water containing 0.1 % TFA through pump A and ACN:MeOH (17:1) through pump B with ratio 10:90, respectively was chosen as it offered the best peak resolution at preset HPLC conditions. The injection volume was 20 µL. Validation of the developed method was performed under the International Conference on Harmonization guidelines and Guidance for Industry: Bioanalytical Method Validation [21,22]. The validation was included an evaluation of the following parameters: linearity, selectivity, sensitivity, accuracy and precision. Validation and stability studies were carried out against spiked plasma samples. Briefly, a 10 µL of a sample from workings solutions was vortexed with 90 µL of blank plasma to prepare dilutions equivalent to 8, 16, 32, 64, 128, 256 and 400 µg/mL. The drug was extracted from plasma as described in "bio-distribution study" section and evaluated on HPLC, accordingly.

The detector response was found linear against the concentration range 5-160 µg/mL of OA in methanol under specific chromatographic condition ($y = 93308x - 79018$, $R^2 = 0.999$). The retention time (T_r) of OA was observed at 12 ± 1 min at a total run time of 15 min (chromatograms not shown). A linear calibration curve ($y = 15509x - 114429$, $R^2 = 0.9981$) was obtained against spiked plasma concentrations (5-160 µg/mL). The developed method was further validated against spiked plasma samples under strict compliance as we did previously [23,24] and all the results were tabulated (**Table S1**).

Only the quantifiable samples, except the lowest quantifiable concentration (LLOQ = 5 µg/mL), were subjected to validation. Efficient recovery of OA was attained (> 90%). The relative standard deviation (RSD) below 1% indicated the high sensitivity of the developed method while coefficient of variance (% CV) less than 10 at all the observed concentrations against stated concentrations justified that the method was reliable enough to apply for bioavailability study. It inferred that the OA was stable in matrix system as there were no significant differences among the concentrations of inter-day analysis (**Table S1**).

Table S1. Validation of HPLC method for spiked plasma samples of OA under strict compliance.

**Parameters**	**Test Applied**	**Treated Samples**	**Test Conditions**	**Statistical Tool**	**Results**	**Acceptance Criteria**	**Remarks**
System Suitability	Retention Time (T_r)	*n*> 3	Normal and accelerated stability study	-	$\overline{X}$ ± SEM, % RSD (11.87 ± 0.12, 1.77)	% RSD ≤ 2	No interfering peaks were observed in quality and plasma samples
System Performance	Recovery	*n*> 3	Predefined	$\%\ \text{Recovery} = *(A_{ext.}/A_{spiked}) \times 100$	95–103%	95–105%	Efficient extraction of the drug was done
Selectivity and Linearity	Goodness of Fit Test	*n*> 3	Predefined	-	y = 15509x – 114429, $r^2 = 0.9981$	$r^2 < 0.9$	Results were reproducible at the stated range
LOD and LOQ (μg/mL)	Qualification and Quantification	*n*> 6	Predefined	-	$\overline{X}$ ± SEM, % RSD (19.33 ± 2.85, 0.51 and 32.03 ± 0.15, 0.79)	% RSD ≤ 1	-
Accuracy (%)		*n*> 3	Predefined	$\%\ \text{Accuracy} = **(C_{obs.mean}/C_{nom.}) \times 100$	99.32-102.88 (%), 0.45–3.2%	% CV < 10	Developed HPLC method was reliable
Precision (% CV)	Intra-day and Inter-day variations	*n*> 3	$p<0.05$	Un-paired t-test followed by Mann Whitney test	ns ($p = 0.8983$)	% CV < 10	Validated results

*$A_{ext.}$ = Amount extracted, A_{spiked} = amount spiked; ** $C_{obs.mean}$ = Mean observed concentration, $C_{nom.}$ = Nominal concentration.
